# Genetically-determined body mass index and the risk of atrial fibrillation progression in men and women

**DOI:** 10.1371/journal.pone.0246907

**Published:** 2021-02-18

**Authors:** J. E. Siland, B. O. Nguyen, R. R. de With, I. C. Van Gelder, P. van der Harst, M. Rienstra

**Affiliations:** 1 Department of Cardiology, University of Groningen, University Medical Center Groningen, Groningen, The Netherlands; 2 Department of Genetics, University of Groningen, University Medical Center Groningen, Groningen, The Netherlands; 3 Department of Heart and Lungs, University Medical Center Utrecht, University of Utrecht, Utrecht, The Netherlands; Ohio State University, UNITED STATES

## Abstract

**Aims:**

Limited causal evidence is available on the relationship between body mass index (BMI) and atrial fibrillation (AF) progression. Sex differences have been noted and may be relevant for AF progression. We investigated the association between the BMI Genetic Risk Score (GRS) and AF progression in men and women of the Groningen Genetic Atrial Fibrillation (GGAF) cohort.

**Methods and results:**

The GGAF cohort (n = 2207) is a composite of 5 prospective cohorts with individuals of European ancestry. AF patients with genetic information, with at least 12 months follow-up and AF progression data were included. AF progression was defined as progression from paroxysmal to persistent/permanent AF, or persistent to permanent AF. A BMI GRS was constructed of genetic variants associated with BMI. Univariate and multivariate Cox proportional hazard regression analyses were performed in the total population and in men and women, separately. During a median follow-up of 34 [interquartile range 19–48] months 630 AF patients (mean age 62±11, 36% women, BMI of 28±5) were analyzed, and men and women developed similar AF progression rates (respectively 6.5% versus 6.1%). The BMI GRS was not associated with AF progression either as a continuous variable or in tertiles in the overall population. However, the BMI GRS was associated with the tertile of the highest BMI GRS in women (n = 225), also after multivariable adjustments of clinical risk factors (Hazard ratio 2.611 (95% confidence interval 1.151–5.924) p = 0.022).

**Conclusions:**

Genetically-determined BMI is only associated with women at risk of AF progression. The results may be supporting evidence for a causal link between observed BMI and AF progression in women. We emphasize the need for further investigation of genetically determined BMI and observed BMI to optimize AF management in women with increased risk for AF progression.

## Introduction

Obesity and atrial fibrillation (AF) are both emerging global epidemics and causing a growing economic burden [1]. AF is associated with an increased risk of cardiovascular diseases like heart failure, stroke, dementia and death [1]. When AF progresses from paroxysmal / self-limiting to more sustained / non-self-limiting forms of AF, more cardiovascular events occur [2]. In an attempt to unravel the development of AF progression, several risk factors were identified. Higher observed body mass index (BMI) has been associated with an increased risk of AF. Previously, the association between observed BMI and the risk of incident AF differed between men and women [3, 4]. Albeit contradictory findings, it is possible that sex is also a modifying factor in the relation between increased observed BMI and development or AF progression.

An increased observed BMI is one of the risk factors of AF progression [5]. One of the explanations is that an increased observed BMI causes atrial remodeling such as atrial stretch, and diastolic impairment [6]. These effects can be direct consequences of an increased observed BMI or indirectly via e.g. inflammation, sympathetic activation or associated comorbidities, such as hypertension and obstructive sleep apnea, that can induce atrial stretch [6]. However, evidence that an increased observed BMI causes AF progression is still limited.

Genetic variants associated with observed BMI could help to further elucidate its role in AF progression. If an association between genetic variants of BMI and AF progression exists, it may support the notion of a causal link. Therefore, we investigate the association between genetically-determined BMI and AF progression in men and women with short-lasting AF in the Groningen Genetic Atrial fibrillation (GGAF) cohort.

## Methods

### Study population

**Fig 1:**
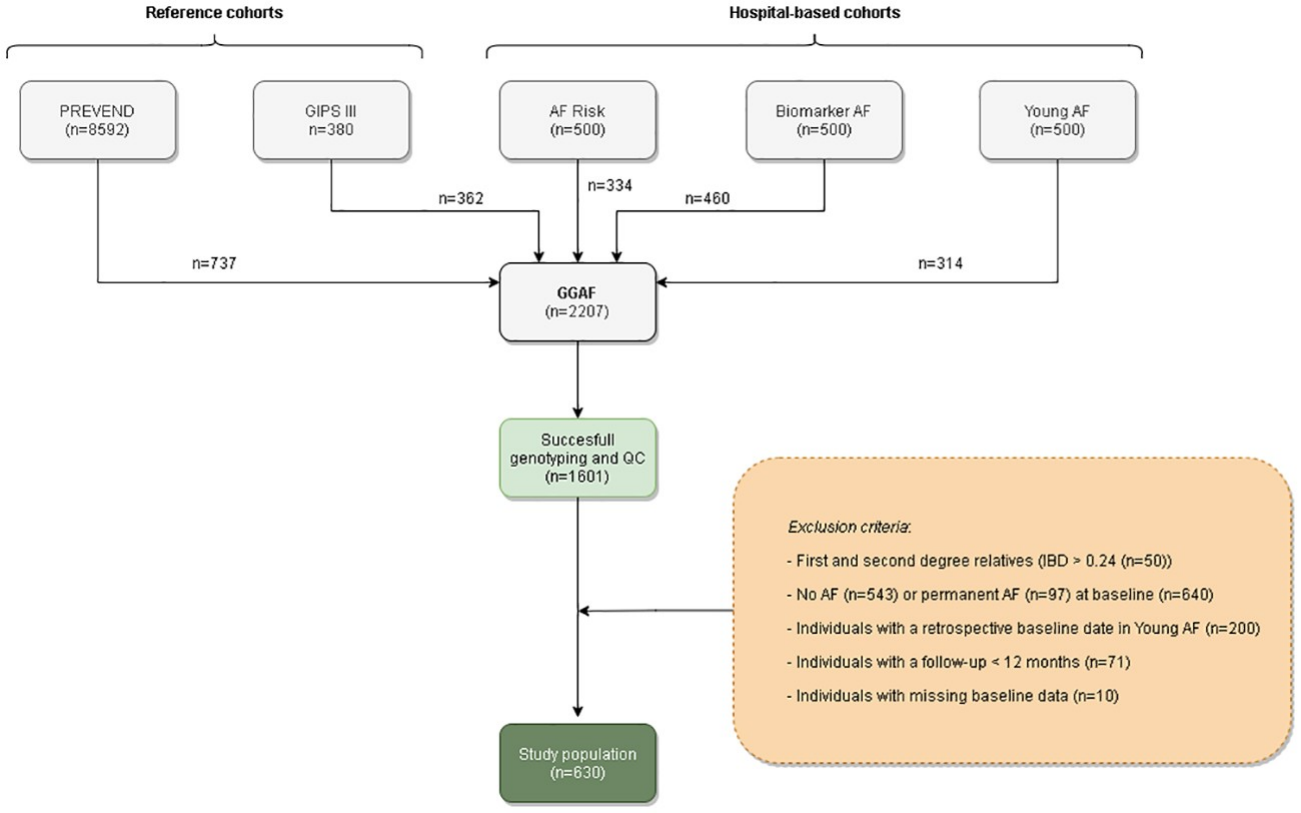
Flowchart of the selected study population. Abbreviations: AF = atrial fibrillation, AF Risk = AF Risk study, Biomarker AF = Biomarker AF study, GGAF = Groningen Genetic Atrial Fibrillation study, GIPS III = glycometabolic intervention as adjunct to primary percutaneous coronary intervention in ST elevation myocardial infarction III study, IBD = identity by descent, PREVEND = Prevention of Renal and Vascular End-stage Disease study, QC = Quality control, Young AF = Young AF study.

Three hospital-based, prospective, AF registries (AF-RISK study, Biomarker-AF study and Young-AF study) recruited in the University Medical Center Groningen and two age- and sex-matched reference cohorts (Prevention of Renal and Vascular End-stage Disease (PREVEND) study and the Glycometabolic Intervention as adjunct to primary Percutaneous coronary intervention in ST elevation myocardial infarction (GIPS) III) are enclosed in the GGAF cohort (n = 2207). The study protocols of the above-mentioned studies are in line with the Declaration of Helsinki, were approved by the local institutional review board (Medisch Ethische Toetsingscommissie Universitair Medisch Centrum Groningen), and written informed consent was received of all included individuals. A detailed description of participating cohorts was previously published [7–9]. In our study we selected AF patients of European descent with prospectively registered AF progression, successful genotyping and a follow-up of at least 12 months and with a maximum of 60 months in the AF-RISK study, Biomarker-AF study and Young-AF study. Individuals without AF at baseline, permanent AF or missing data at baseline were excluded (Fig 1).

### Genotyping, quality control and imputation

The GGAF cohort was genotyped as part of the Broad AF Study (Broad AF) at the Broad Institute [9]. The Infinium PsychArray-24 v1.2 Bead Chip was used and GenomeStudio v1.6.2.2 and Birdseed v1.33 were used to call common variants (≥ 0.5% minor allele frequency (MAF)). In brief, pre-imputation quality control filtering of samples and variants was conducted. Samples were filtered based on completeness (>97% sample call rate), heterozygosity (>± 0.2), genetic ancestry outliers and related first- and second-degree AF patients (Identity By Descent < 0.24) to avoid bias. Variants were excluded when clear deviations from the Hardy-Weinberg proportions (HWE) (P-value in references > 1 x 10–6) existed, high degree of missingness (<0.98) or low minor allele frequency (<0.005). Imputation was performed with HRC reference v1.1 panel on the Michigan Imputation Server v1.0.1 using Minimac. Further detailed information is described previously [9]. Only AF patients with sufficient quality control and imputation were included in current analyses.

### BMI Genetic Risk Score

A Genetic Risk Score was constructed of 941 genetic variants associated with BMI available from previous meta-analysis of Yengo and colleagues [10], and calculated for every AF patient by summing the dosage of each BMI risk allele weighted by the natural logarithm of the relative risk for each genetic variant. Thus, the effect size of every single genetic variant associated with BMI was summed into a Genetic Risk Score value for each AF patient, conditional of the relative risk for each genetic variant. The higher the BMI Genetic Risk Score in the AF patient, the higher the effect size of genetic variants associated with BMI present in the genotyped data of the AF patient. The Genetic Risk Score was constructed using the open source whole genome association data analyses toolset PLINK [11].

### Definitions

The AF diagnosis was based on the documentation of a 12-lead electrocardiogram (ECG), and was confirmed by a cardiologist. The AF type at baseline was determined by the information documented in the medical records. In line with the 2016 European Society of Cardiology AF guidelines the following types are distinguished: paroxysmal AF (≤ 7 consecutive days of AF), persistent AF (> 7 consecutive days of non-self-terminating AF) and permanent AF (conversion to sinus rhythm failed and cannot be restored and/or is no longer pursued by the treating cardiologist) [12]. Heart failure was defined as the presence of New York Heart Association functional Class II or III, previous hospitalization for heart failure or left ventricular ejection fraction (LVEF) ≤ 45%. Hypertension was determined by a systolic blood pressure > 140 mmHg, diastolic blood pressure > 90mmHg, or by use of antihypertensive drugs. By dividing the weight to height squared (kg/m^2^) observed BMI was calculated. Overweight was defined as observed BMI ≥ 25 kg/m^2^, and obesity was defined as observed BMI ≥ 30 kg/m^2^. Diabetes was defined as type I or type II diabetes with use of anti-diabetic drugs. Peripheral artery disease was defined by a clinical diagnosis of a vascular specialist or observed with Doppler ultrasonography. The clinical diagnosis of stroke or transient ischemic attack (TIA) and chronic obstructive pulmonary disease (COPD), and myocardial infarction were obtained from the medical records.

### Follow-up data of AF progression

AF progression was defined as paroxysmal AF that developed into persistent or permanent AF, or persistent AF that developed into permanent AF. The time to AF progression was the time to development of persistent AF or electrical cardioversion or permanent AF. Follow-up visits were planned 3 monthly during the first year, thereafter yearly. If AF patients visited the treating cardiologist in the meantime, information from the medical records was collected and change of AF type was noted. Treatment after inclusion in the registry was not specified in the study protocols and led to discretion of the treating physician. The follow-up period started at the inclusion date (first inclusion November 2009) and was continued until the most sustained type of AF progression, the last contact date (last patient December 2017), or until death, with a maximum follow-up duration of 60 months.

### Statistical analyses

The description of the statistics of the study population and separate analyses were presented as mean (standard deviation (SD)) or median [interquartile range] for continuous variables, depending on the normality of data. Categorical data were presented as percentages and numbers. Chi-squared test was performed for categorical data and a t-test for continuous data. Cox proportional hazard regression analyses were used to find determinants of AF progression. In case of no progression, time until death or last contact date was used as time-to-event. Principal components of the genotype matrix of GGAF cohort were calculated to correct for population stratification. Results are given as hazard ratios (HR) with 95% confidence interval (CI). Analyses were performed for men and women separately. Univariate Cox proportional hazard regression was used to find determinants of AF progression. The multivariable Cox proportional hazard regression was adjusted for age, sex and 2 principal components determined by a scree plot (S1 Fig), AF type at baseline, time of follow-up, hypertension, age > 75 years, TIA or stroke, COPD, heart failure, diabetes, myocardial infarction, and peripheral vascular disease [13, 14]. Only variables with p<0.05 were considered statistically significant. Schoenfeld residuals were evaluated to test the proportional hazard assumption. Statistical analyses were performed using IBM SPSS Statistics for Windows, version 23.0 (IBM Corp., Armonk, N.Y., USA), R (version 3.1.6; R Foundation for Statistical Computing, Vienna, Austria) and PLINK [11].

## Results

### Patient characteristics

**Fig 2:**
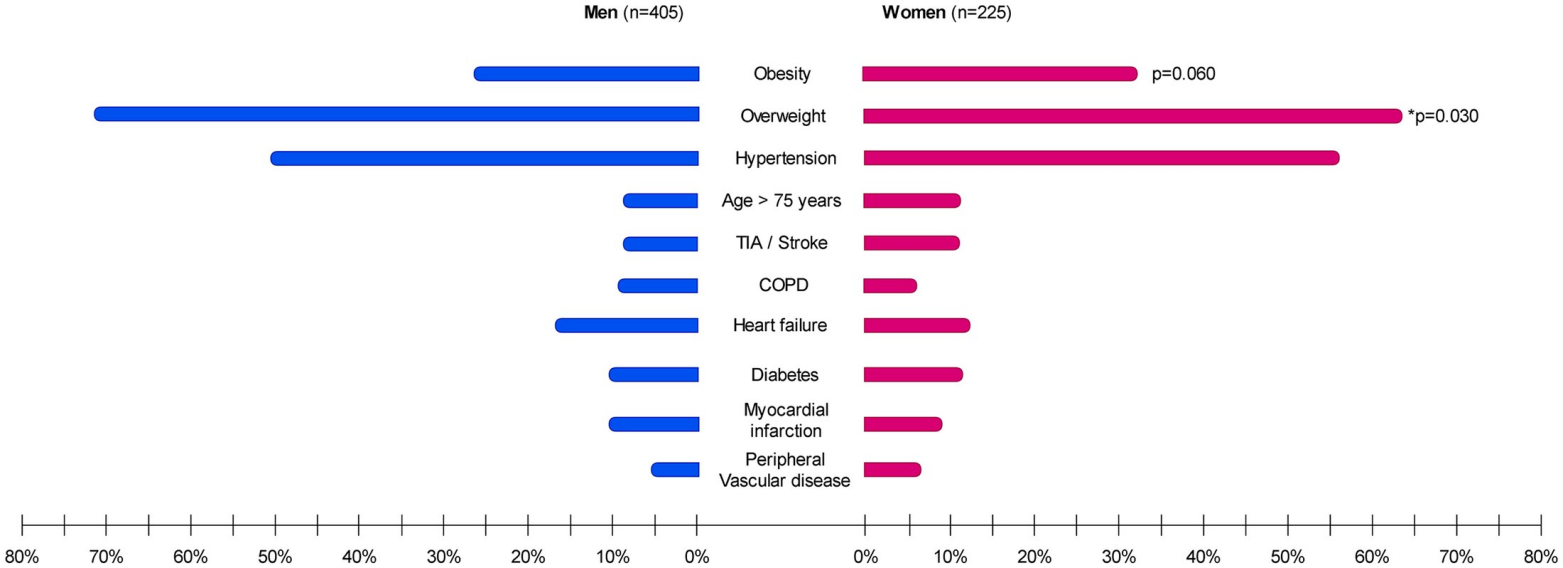
Sex-differences in clinical characteristics. Results of Chi-squared test p < 0.1 are given. The asterisk (*) indicates statistical significance (p < 0.05). Abbreviations: COPD = chronic obstructive pulmonary disease, TIA = transient ischemic attack.

Of the 2207 individuals included in the GGAF cohort, we included 630 AF patients with short-lasting paroxysmal or persistent AF in present analysis (Fig 1). At baseline the mean age was 62±11 years, 225 (36%) were women and 496 (79%) AF patients were diagnosed with paroxysmal AF and 134 (21%) AF patients were diagnosed with persistent AF. AF patients had a observed BMI of 28 ± 5, and 180 (29%) AF patients were obese. Two-hundred-twenty-five (36%) women with a mean age of 63 ± 11 years, and 405 (64%) men with mean age of 61 ± 11 years were included. Obesity was not significantly more prevalent in women compared to men (respectively 26% versus 33%, p = 0.060). However, men were more often overweight than women (respectively 73% versus 64%, p = 0.035) (Table 1, Fig 2).

**Table 1:** Characteristics of the AF patients in the GGAF cohort.

	Study population (n = 634)	Women n = 225 (35.7%)	Men n = 405 (64.3%)
**Genetic Risk Score**			
BMI Genetic Risk Score	1.766 ± 0.525	1.749 ± 0.528	1.777 ± 0.524
**Clinical characteristics**			
Age (years)	62 ± 11	63 ± 11	61 ± 11
BMI (kg/m^2^)	28 ± 5	28 ± 5	28 ± 4
Obesity	180 (28.6)	75 (33.3)	105 (25.9)
Overweight	440 (69.5)	144 (64.0)	296 (72.6)
Hypertension	333 (52.9)	127 (56.4)	206 (50.9)
Age > 75 years	63 (10.0)	25 (11.0)	38 (9.0)
TIA or stroke	63 (10.0)	24 (10.7)	39 (9.6)
COPD	49 (7.8)	14 (6.2)	35 (8.6)
Heart failure	101 (16.0)	30 (13.3)	71 (17.5)
Diabetes Mellitus	68 (10.8)	26 (11.6)	42 (10.4)
Myocardial infarction	65 (10.3)	19 (8.4)	46 (11.4)
Peripheral Artery disease	38 (6.0)	12 (5.3)	26 (6.4)

Values are mean ± SD or numbers (percentages). Abbreviations: AF = Atrial Fibrillation, BMI = Body Mass Index, COPD = Chronic Obstructive Pulmonary Disease, SD = standard deviation, TIA = Transient Ischemic Attack.

### BMI Genetic Risk Score

**Fig 3:**
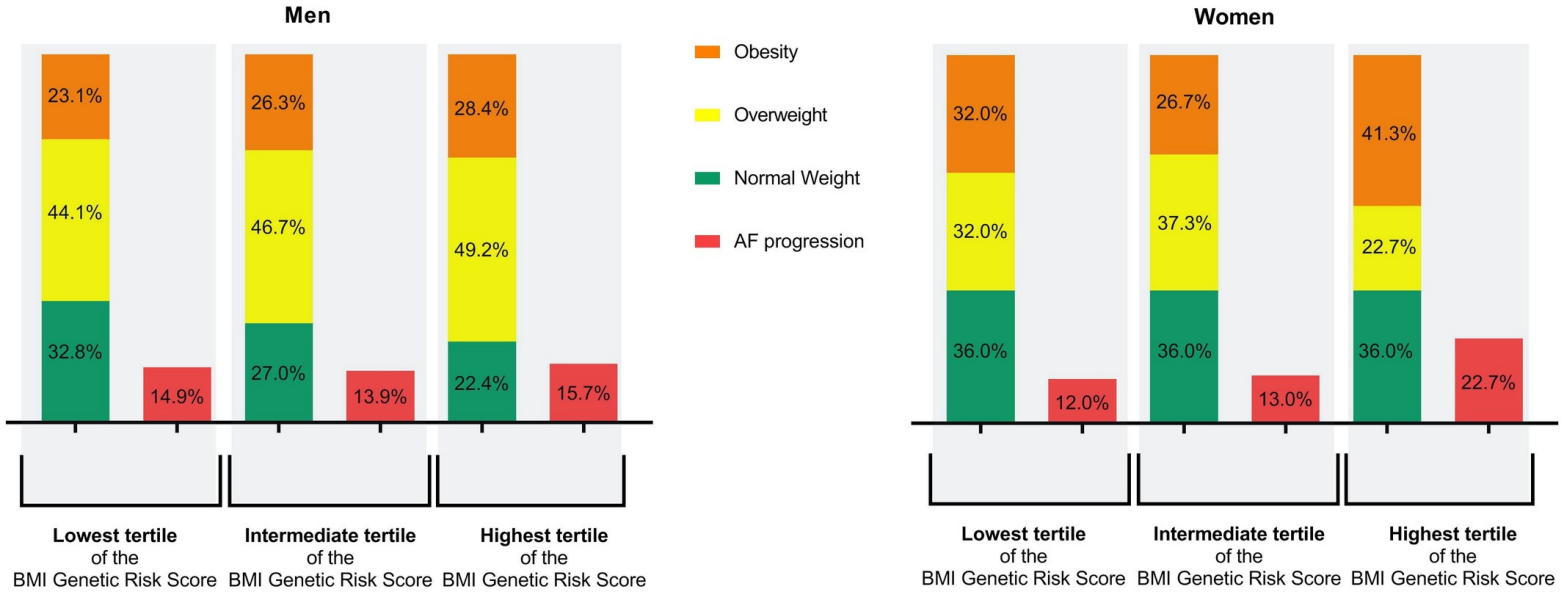
Characteristics of weight and AF progression in BMI Genetic Risk Score tertiles of both men and women. In this figure the distribution of obesity, overweight and normal weight and AF progression in the BMI genetic risk tertiles are displayed in percentages. Abbreviations: AF = atrial fibrillation, BMI = body mass index.

Women with the highest BMI Genetic Risk Score were not significantly more obese than women who did not have the highest BMI Genetic Risk Score (respectively 41% versus 29%, p = 0.099). Likewise, men with the highest BMI Genetic Risk Score were not significantly more obese than men who did not have the highest BMI Genetic Risk Score (respectively 28% versus 25%, p = 0.506) (Fig 3, S1 and S2 Tables).

### Follow up

**Fig 4:**
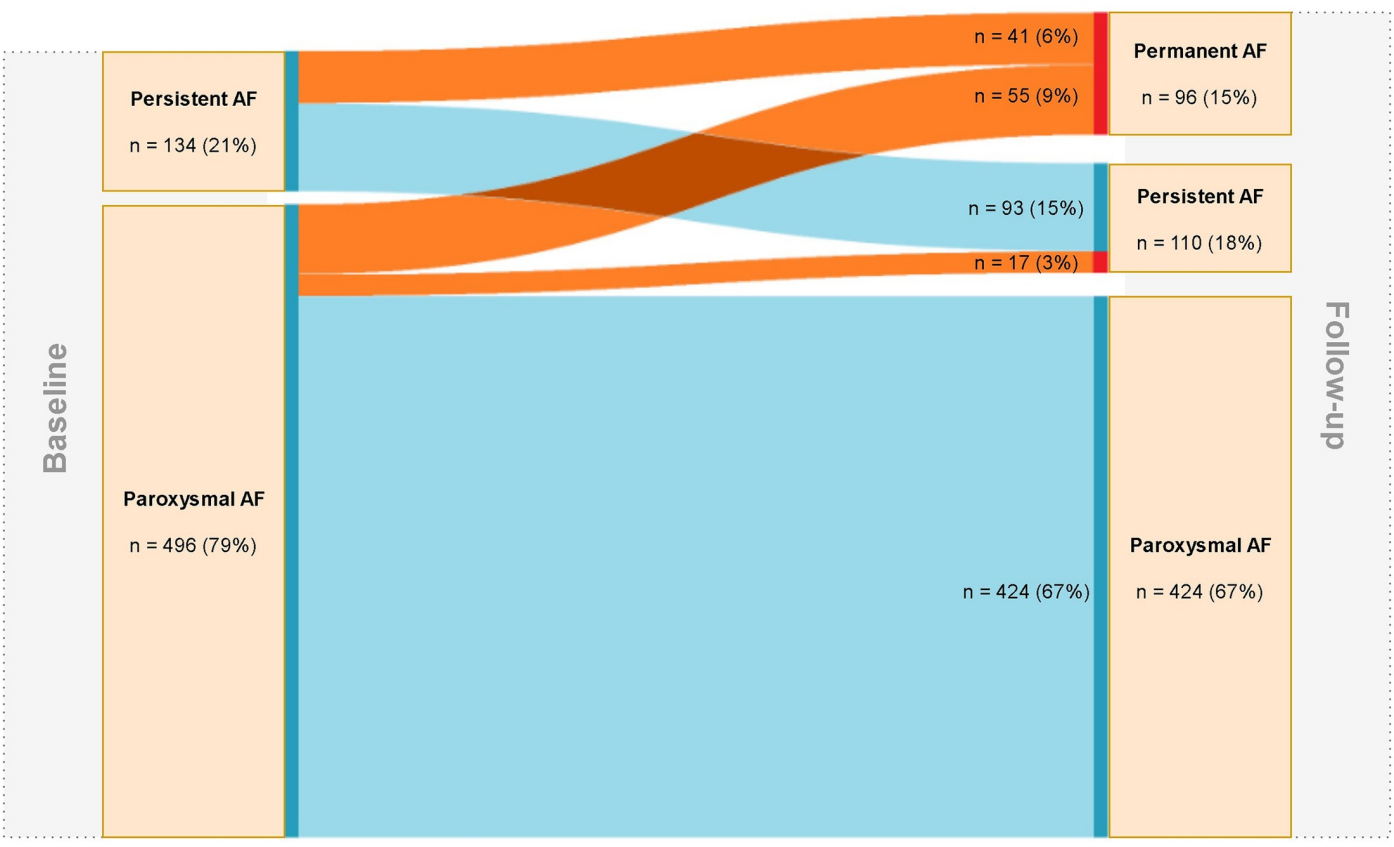
Overview of AF progression in the GGAF cohort. On the left side of Fig 2 AF type and numbers (percentages) at baseline are shown. On the right side of Fig 2 AF type and numbers (percentages) at follow-up are shown. The orange lines represent the transition to AF progression, the blue lines represent no progression. Abbreviations: AF = atrial fibrillation.

During a median follow-up period of 34 [Interquartile range 19–48] months 113 (18%) AF patient developed AF progression. The development of AF to either persistent AF or permanent AF resulted in a total AF progression rate of 6.4% per patient year, and men and women had no significant different yearly AF progression rates (respectively 6.5% versus 6.1%) (Fig 4).

### BMI Genetic Risk Score and AF progression

In univariate Cox proportional hazard regression analyses the BMI Genetic Risk Score as a continuous variable was not associated with AF progression (Hazard ratio (HR) 1.342 (95% confidence interval (CI) 0.953–1.891); p = 0.092). Subsequently, the BMI Genetic Risk Score was divided into tertiles, quartiles and quintiles to observe the trend and reliability of the data when age- and sex adjustments were performed. However, p-values were not larger than p = 0.1, even when correcting for age and sex (S3 Table).

### BMI Genetic Risk Score and AF progression in men and women separately

**Fig 5:**
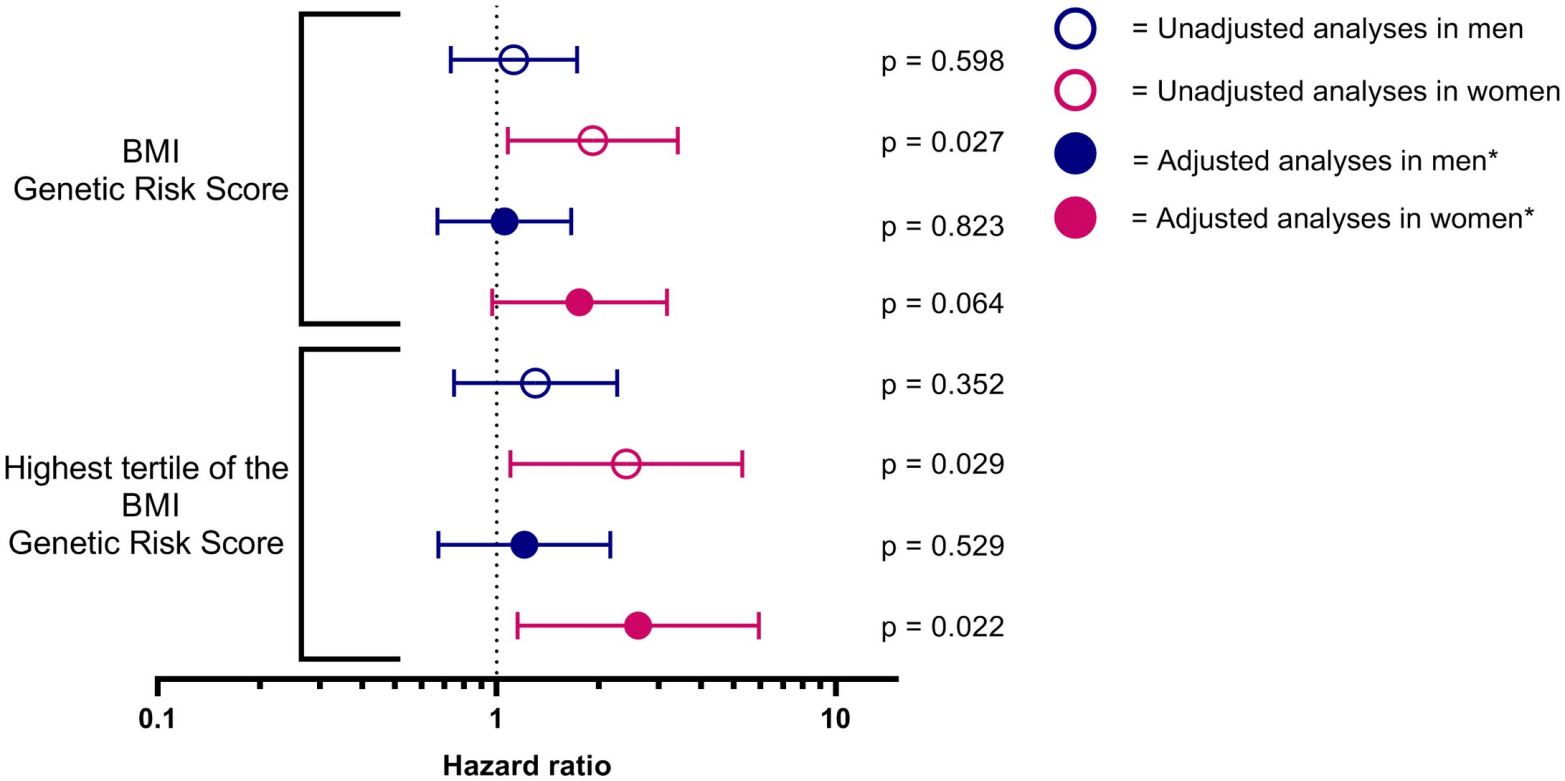
The association between the BMI Genetic Risk Score and AF progression in men and women. *Adjusted for principal components, age at inclusion, AF type at baseline, follow-up duration, BMI, hypertension, age > 75 years, transient ischemic attack or stroke, chronic obstructive pulmonary disease, heart failure, and peripheral vascular disease. Abbreviations: BMI = body mass index.

Univariate Cox proportional hazard regression analyses of the tertile with the highest BMI Genetic Risk Score were significantly associated with AF progression in women (respectively HR 2.409 (95% CI 1.097–5.293) p = 0.029). In contrast, separate univariate Cox proportional hazard regression analyses of the tertile with the highest BMI Genetic Risk Score were not significantly associated with AF progression in men (HR 1.301 (95% CI 0.748–2.266) p = 0.352). In multivariate analyses the significant association between the tertile with highest BMI Genetic Risk Score and AF progression remained statistically significant in women, when adjusted for principal components, age, AF type at baseline, follow-up duration, observed BMI, hypertension, age > 75 years, TIA or stroke, COPD, heart failure, and peripheral vascular disease (HR 2.611 (95% CI 1.151–5.924) p = 0.022) (Fig 5). Addition of antiarrhythmic medication, statins and pulmonary vein isolation in the multivariate analyses did not change our findings (S4 and S5 Tables).

## Discussion

The main findings of our analysis were: 1) Men and women with short-lasting AF had similar AF progression rates in the GGAF cohort, 2) Genetically-determined BMI was associated with AF progression in women and not in men, 3) Our results may be supporting evidence that, in women, observed BMI may cause AF progression.

### Genetically-determined BMI and observed BMI in the GGAF population

Observed BMI ≥ 30 kg/m^2^ increases the risk by approximately 50% for developing AF [15]. On average, the AF patients of the GGAF cohort have an increased observed BMI, which is typical for individuals with AF [2, 13]. The observed BMI distribution in our study is similar to the observed BMI of European AF patients. In the European heart Survey several clinical types of AF were investigated in 5333 AF patients from 35 European countries [16]. The mean observed BMI of the included individuals with paroxysmal AF and persistent AF of European Heart Survey is comparable to our cohort (respectively mean (SD) observed BMI of 28±9 versus 28±5). Moreover, our cohort is similar to the AF population of the hospital-based Standard versus Atrial Fibrillation specific managemenT study (SAFETY), where more women presented obese and men were more overweight [17]. However, genetic predisposition to a high BMI may not be reflected in the observed BMI. More than half the individuals in the highest tertiles of the BMI Genetic Risk Score have an observed BMI ≥ 30 kg/m^2^ (Fig 3). Still, genetically determined BMI may influence the development of AF progression.

### Genetically-determined BMI and AF progression

In a recent systematic review with similar AF progression definitions, 47 studies with over 27 000 AF patients were included and the AF progression rate ranged from 0.8 to 35.6% per patient year. They showed a pooled AF progression rate of 8.2% per patient years in European countries [13]. AF progression in the GGAF cohort was comparable with 6.4% per patient years. The slightly lower AF progression rate in the GGAF cohort is potentially due to the mean age below 65 years. However, the total AF population did not show an association between genetically-determined BMI and AF progression. Absence of an association may be caused by lack of power, but may also be explained by potential differences in underlying mechanisms how obesity causes incident AF versus AF progression. It is possible that genetically-determined BMI is indeed causal to incident AF, but more a mediating factor regarding the progression to non-self-limiting forms of AF.

### Genetically-determined BMI in women

In our cohort separate analyses for men and women showed that women with the highest BMI Genetic Risk were at increased risk of AF progression. Previously, the Woman’s Health Study showed that an increased observed BMI may be of importance in women with AF progression. Women who developed AF progression were more obese compared to women who maintained paroxysmal AF two years after AF diagnosis [18]. In addition, obese women had more left atrial dilatation. In the FAT associated CardiOvasculaR dysfunction (FATCOR) study clinical and echocardiographic data from 581 individuals without cardiovascular disease and observed BMI > 27 kg/m^2^ were analyzed [19]. Women had a higher prevalence of left atrial dilatation than men, which suggests that there may be sex-specific differences in atrial remodeling and underlying comorbidities that eventually could results in AF development or progression [20]. However, the role of obesity in the development of AF progression in women remains largely uninvestigated.

### Clinical perspective

Unveiling the impact of genetically-determined BMI and sex on AF progression may partly explain the existence of inter-individual differences in risk of AF progression. Our results using genetically-determined BMI as proxy for observed BMI / obesity, suggest that observed BMI may be of more importance in women than men regarding risk of AF progression. In the current European guidelines weight loss, combined with risk factor management is recommended [12]. Weight control may minimize their risk of AF progression more in women than in men. However, longitudinal observations of body mass index were not available in our cohort and clinical trials are warranted.

### Strengths and limitations

Strengths of our study are the use of both genetic and phenotypic information to unravel the potential causal role of observed BMI to AF progression. AF patients of the GGAF cohort are deeply phenotyped, and AF ascertainment and AF progression types were defined according to the European guidelines [12]. Compared to prior studies of genetically-determined BMI and incident AF, the BMI Genetic Risk Score in our study has even more predictive power [10]. The increased amount of discovered associated genetic variants improves determination of genetic predisposition. Unfortunately, our sample size was not sufficient to perform Mendelian randomization analyses. However, we hope our results will encourage researchers in the field of AF to collect large scale data of genetics and AF progression and enable Mendelian randomization analyses in the future. Albeit that more information is needed to explain the effects of genetics on AF progression, clinicians and researchers may consider the findings of our study a pilot for research in the field of genetics and AF progression. Additionally, several other limitations need to be considered. Firstly, we did not perform continuous rhythm monitoring, and relied on intermittent ECGs to diagnose AF progression, this may have led to misclassification. Asymptomatic AF episodes may have been missed and concealed the time of AF progression. Secondly, one component of obesity is genetics, another component of obesity is lifestyle. We stress that important lifestyle risk factors of AF progression, such as physical activity, fitness and alcohol intake were not investigated in our cohort [21, 22]. Adjustments for all environmental confounders of observed BMI could not possibly be made in our analyses. However, despite the fact that the used genetic variants are estimated to explain only 6% of observed BMI variance [10], the association between genetically-determined BMI and AF progression maintained in women. Thirdly, heritability and genetic effects of BMI genetic variants could potentially be stronger in women, like genetic variants associated with waist-hip-ratio [23]. A stronger genetic effect of BMI genetic variants in women could have caused the significant association between BMI Genetic Risk Score and AF progression in women. Fourthly, by all means, the results should be interpreted with caution since a relatively small sample size was used, and the men-women ratio in this study (2:1) limits sex-specific analyses. Additionally, albeit that the use of antiarrhythmic medication, statins or pulmonary vein isolation did not change our findings fundamentally, no data of change in medication during follow-up was available. Furthermore other confounding factors may play a role. Further investigation of BMI genetic risk, AF progression and confounding factors should be conducted. Moreover, we would like to stress that it is important to replicate our results in independent cohorts across race-ethnicity to ensure the reliability of our findings. Finally, our results are not generalizable to all populations, only to individuals with short-lasting AF from European ancestry.

## Conclusions

In the GGAF cohort men and women with short-lasting AF had similar AF progression rates. However, genetically-determined BMI was only associated with women at risk of AF progression, independent from clinical risk factors, including observed BMI. The association between genetically-determined BMI and AF progression was not present in men. Our study may provide supporting evidence that observed BMI may be causally linked to AF progression in women. However, the results should be interpreted with caution. The causality of observed BMI to AF progression and optimization of AF management should be investigated in other deep phenotyped AF populations and clinical trials considering genetically-determined BMI and intervention of the observed BMI.

## Supporting information

S1 Fig Scree plot of 10 principal components in the GGAF cohort. (DOCX)

S1 Table Baseline characteristics of women divided into tertiles of the BMI Genetic Risk Score. (DOCX)

S2 Table Baseline characteristics of men divided into tertiles of the BMI Genetic Risk Score. (DOCX)

S3 Table Cox regression analyses with BMI Genetic Risk Score and AF progression. (DOCX)

S4 Table Medication at baseline. (DOCX)

S5 Table Multivariate regression in women. (DOCX)
