# Supplementary material for: Genetically-determined body mass index and the risk of atrial fibrillation progression in men and women
Source: PLoS One. 2021 Feb 18;16(2):e0246907. doi: 10.1371/journal.pone.0246907 (PMC7891778; doi:10.1371/journal.pone.0246907)
Supplement: S1 Fig — (DOCX) [file pone.0246907.s001.docx]

**S1 Fig.** Scree plot of 10 principal components in the GGAF cohort.


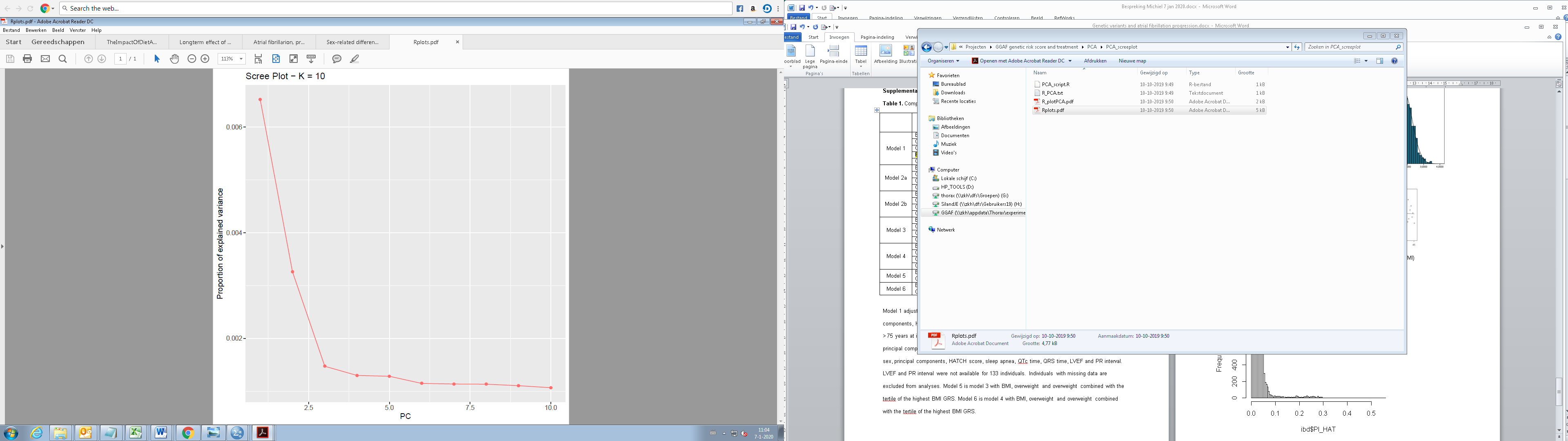


Ten principal components in the GGAF cohort are shown in the scree plot. The point where the slope of the scree plot is leveling off indicates the number of the eigenvalue factors that should be retained. Abbreviations: PC = Principal components.
