# Supplementary material for: Genetically-determined body mass index and the risk of atrial fibrillation progression in men and women
Source: PLoS One. 2021 Feb 18;16(2):e0246907. doi: 10.1371/journal.pone.0246907 (PMC7891778; doi:10.1371/journal.pone.0246907)
Supplement: S1 Table — (DOCX) [file pone.0246907.s002.docx]

**S1 Table.** Baseline characteristics of women divided into tertiles of the BMI Genetic Risk Score.

|  | **Total study population**  **(n=225)** | **Lowest tertile of BMI GRS**  **(n=75)** | **Intermediate tertile of BMI GRS**  **(n=75)** | **Highest tertile of BMI GRS**  **(n=75)** |
| --- | --- | --- | --- | --- |
| **Genetic Risk Score** | | | | |
| BMI Genetic Risk Score | 1.749 ± 0.528 | 1.189 ± 0.280 | 1.721 ± 0.147 | 2.337 ± 0.274 |
| **Clinical characteristics** | | | | |
| Age (years) | 63.4 ± 11.4 | 63.3 ± 12.0 | 63.5 ± 11.5 | 63.4 ± 10.6 |
| BMI (kg/m^2^) | 28 ± 5 | 28 ± 6 | 27 ± 5 | 28 ± 5 |
| Obesity | 75 (33.3) | 24 (32.0) | 20 (26.7) | 31 (41.3) |
| Overweight | 144 (64.0) | 48 (64.0) | 48 (64.0) | 48 (64.0) |
| Hypertension | 127 (56.4) | 46 (61.3) | 41 (54.7) | 40 (53.3) |
| TIA or stroke | 24 (10.7) | 10 (13.3) | 5 (6.7) | 9 (12.0) |
| COPD | 14 (6.2) | 4 (5.3) | 3 (4.0) | 7 (9.3) |
| Heart failure | 30 (13.3) | 9 (12.0) | 4 (5.3) | 17 (22.7) |
| Diabetes | 26 (11.6) | 8 (10.7) | 8 (10.7) | 10 (13.3) |
| Myocardial infarction | 19 (8.4) | 9 (12.0) | 3,9 (5.3) | 6 (8.0) |
| Peripheral artery disease | 12 (5.3) | 5 (6.7) | 2 (2.7) | 5 (6.7) |

Values are mean (SD), numbers (percentages, median for categorical data and (interquartile range) for continuous variables. Abbreviations: AF = Atrial Fibrillation, BMI = Body Mass Index, COPD = Chronic Obstructive Pulmonary Disease, GRS= Genetic Risk Score, SD = standard deviation, TIA = Transient Ischemic Attack.
