# Supplementary material for: Genetically-determined body mass index and the risk of atrial fibrillation progression in men and women
Source: PLoS One. 2021 Feb 18;16(2):e0246907. doi: 10.1371/journal.pone.0246907 (PMC7891778; doi:10.1371/journal.pone.0246907)
Supplement: S2 Table — (DOCX) [file pone.0246907.s003.docx]

**S2 Table.** Baseline characteristics of men divided into tertiles of the BMI Genetic Risk Score.

|  | **Total study population**  **(n=405)** | **Lowest tertile of BMI GRS**  **(n=134)** | **Intermediate tertile of BMI GRS**  **(n=137)** | **Highest tertile of BMI GRS**  **(n=134)** |
| --- | --- | --- | --- | --- |
| **Genetic Risk Score** | | | | |
| BMI Genetic Risk Score | 1.777 ± 0.524 | 1.210 ± 0.275 | 1.774 ± 0.134 | 2.347 ± 0.297 |
| **Clinical characteristics** | | | | |
| Age (years) | 60.7 ± 11.2 | 60.1 ± 12.8 | 61.3 ± 10.1 | 60.7 ± 10.6 |
| BMI (kg/m^2^) | 28 ± 4 | 27 ± 5 | 28 ± 4 | 28 ± 5 |
| Obesity | 105 (25.9) | 31 (23.1) | 36 (26.3) | 38 (28.4) |
| Overweight | 294 (72.6) | 90 (67.2) | 100 (73.0) | 104 (77.6) |
| Hypertension | 206 (50.9) | 62 (46.3) | 67 (48.9) | 77 (57.5) |
| TIA or stroke | 39 (9.6) | 13 (9.7) | 9 (6.6) | 17 (12.7) |
| COPD | 35 (8.6) | 12 (9.0) | 10 (7.3) | 13 (9.7) |
| Heart failure | 71 (17.5) | 26 (19.4) | 22 (16.1) | 23 (17.2) |
| Diabetes | 42 (10.4) | 10 (7.5) | 14 (10.2) | 18 (13.4) |
| Myocardial infarction | 46 (11.4) | 18 (13.4) | 16 (11.7) | 12 (9.0) |
| Peripheral artery disease | 26 (6.4) | 15 (11.2) | 5 (3.7) | 6 (4.5) |

Values are mean (SD) or numbers (percentages) median for categorical data and (interquartile range) for continuous variables. Abbreviations: AF = Atrial Fibrillation, BMI = Body Mass Index, COPD = Chronic Obstructive Pulmonary Disease , GRS= Genetic Risk Score, SD = standard deviation, TIA = Transient Ischemic Attack.
