# Supplementary material for: Genetically-determined body mass index and the risk of atrial fibrillation progression in men and women
Source: PLoS One. 2021 Feb 18;16(2):e0246907. doi: 10.1371/journal.pone.0246907 (PMC7891778; doi:10.1371/journal.pone.0246907)
Supplement: S3 Table — (DOCX) [file pone.0246907.s004.docx]

**S3 Table.** Cox regression analyses with BMI Genetic Risk Score and AF progression.

| **Characteristics** | **Hazard ratio** | **95% Confidence Interval** | **P-value** |
| --- | --- | --- | --- |
| **Genetic Risk Score** |  |  |  |
| BMI Genetic Risk Score | 1.354 | (0.959 – 1.912) | 0.085 |
| Tertile 1 of the BMI Genetic Risk Score | Reference | Reference | Reference |
| Tertile 2 of the BMI Genetic Risk Score | 1.097 | (0.6741 – 1.785) | 0.710 |
| Tertile 3 of the BMI Genetic Risk Score | 1.555 | (0.9897 – 2.443) | 0.056 |
| Quartile 1 of the BMI Genetic Risk Score | Reference | Reference | Reference |
| Quartile 2 of the BMI Genetic Risk Score | 1.451 | (0.827 – 2.544) | 0.194 |
| Quartile 3 of the BMI Genetic Risk Score | 1.372 | (0.779 – 2.415) | 0.274 |
| Quartile 4 of the BMI Genetic Risk Score | 1.693 | (0.986 – 2.908) | 0.057 |
| Quintile 1 of the BMI Genetic Risk Score | Reference | Reference | Reference |
| Quintile 2 of the BMI Genetic Risk Score | 1.538 | (0.817 – 2.896) | 0.183 |
| Quintile 3 of the BMI Genetic Risk Score | 1.511 | (0.798 – 2.861) | 0.205 |
| Quintile 4 of the BMI Genetic Risk Score | 1.395 | (0.733 – 2.657) | 0.311 |
| Quintile 5 of the BMI Genetic Risk Score | 1.749 | (0.946 – 3.233) | 0.075 |
| **Genetic Risk Score adjusted for age and sex** |  |  |  |
| BMI Genetic Risk Score | 1.309 | (0.925 – 1.851) | 0.129 |
| Tertile 1 of the BMI Genetic Risk Score | Reference | Reference | Reference |
| Tertile 2 of the BMI Genetic Risk Score | 1.072 | (0.658 – 1.746) | 0.780 |
| Tertile 3 of the BMI Genetic Risk Score | 1.523 | (0.968 – 2.396) | 0.069 |
| Quartile 1 of the BMI Genetic Risk Score | Reference | Reference | Reference |
| Quartile 2 of the BMI Genetic Risk Score | 1.380 | (0.786 – 2.423) | 0.263 |
| Quartile 3 of the BMI Genetic Risk Score | 1.281 | (0.726 – 2.261) | 0.392 |
| Quartile 4 of the BMI Genetic Risk Score | 1.654 | (0.962 – 2.844) | 0.067 |
| Quintile 1 of the BMI Genetic Risk Score | Reference | Reference | Reference |
| Quintile 2 of the BMI Genetic Risk Score | 1.461 | (0.776 – 2.751) | 0.241 |
| Quintile 3 of the BMI Genetic Risk Score | 1.412 | (0.745 – 2.674) | 0.291 |
| Quintile 4 of the BMI Genetic Risk Score | 1.357 | (0.712 – 2.588) | 0.354 |
| Quintile 5 of the BMI Genetic Risk Score | 1.654 | (0.916 – 3.131) | 0.093 |

Data of Cox regression of the tertiles, quartiles and quintiles of the BMI Genetic Risk Score and AF progression are shown, where the ascending numbers are coherent with increasing BMI Genetic Risk Score. Abbreviations: AF = Atrial Fibrillation, BMI = Body Mass Index*.*
