# Supplementary material for: Genetically-determined body mass index and the risk of atrial fibrillation progression in men and women
Source: PLoS One. 2021 Feb 18;16(2):e0246907. doi: 10.1371/journal.pone.0246907 (PMC7891778; doi:10.1371/journal.pone.0246907)
Supplement: S4 Table — (DOCX) [file pone.0246907.s005.docx]

**S4 Table.** Medication at baseline.

|  | **All  n=630** | **Men  n=405** | **Women n=225** | **p-value** |
| --- | --- | --- | --- | --- |
| **Use of class I antiarrhythmic medication** | 85 (13.5) | 59 (14.6) | 26 (11.6) | 0.348 |
| **Use of beta blockers** | 388 (62.0) | 238 (58.9) | 150 (66.7) | 0.067 |
| **Use of class III antiarrhythmic medication** | 98 (15.6) | 66 (16.3) | 32 (14.2) | 0.566 |
| **Use of calcium antagonists** | 159 (25.2) | 102 (25.2) | 57 (25.4) | 1.000 |
| **Use of statins** | 241 (38.3) | 162 (40.0) | 79 (35.1) | 0.261 |
| **Pulmonary vein ablation** | 65 (10.4) | 49 (12.2) | 16 (7.21) | 0.070 |

Values are numbers(percentages). Class I antiarrhythmic medication includes flecainide, propafenone, disopyramide, ritmoforine. Class III antiarrhythmic medication includes sotalol (dosage more than 160mg per day) and amiodaron. Calcium antagonist includes verapamil and diltiazem use. Pulmonary vein ablation at baseline is defined as a pulmonary vein ablation maximal 365 days before and maximal 14 days after the baseline visit.
