# Supplementary material for: Genetically-determined body mass index and the risk of atrial fibrillation progression in men and women
Source: PLoS One. 2021 Feb 18;16(2):e0246907. doi: 10.1371/journal.pone.0246907 (PMC7891778; doi:10.1371/journal.pone.0246907)
Supplement: S5 Table — (DOCX) [file pone.0246907.s006.docx]

**S5 Table.** Multivariate regression in women.

| **Adjustments** | **Significant variables** | **Hazard ratio (95% confidence interval)** | **p-value** |
| --- | --- | --- | --- |
| **Model 1** | AF type at baseline (persistent AF) | 2.78 (1.29 – 6.01) | 0.009 |
|  | Highest BMI genetic risk score tertile | 2.59 (1.13 – 5.96) | 0.025 |
|  | Age at inclusion | 1.06 (1.01 – 1.12) | 0.027 |
| **Model 1 + antiarrhythmic medication** | Highest BMI genetic risk score tertile | 2.71 (1.13 – 6.50) | 0.025 |
|  | AF type at baseline (persistent AF) | 2.31 (1.05 – 5.06) | 0.037 |
| **Model 1 + antiarrhythmic medication + statins** | Highest BMI genetic risk score tertile | 2.71 (1.13 – 6.50) | 0.026 |
|  | AF type at baseline (persistent AF) | 2.32 (1.06 – 5.09) | 0.036 |
| **Model 1 + antiarrhythmic medication + statins + PVI** | Highest BMI genetic risk score tertile | 2.61 (1.07 - 6.37) | 0.036 |
|  | AF type at baseline (persistent AF) | 2.44 (1.09 – 5.49) | 0.031 |
|  | BMI | 1.07 (1.00 – 1.13) | 0.037 |

In model 1 adjustments are made for principal components, age at inclusion, AF type at baseline, follow-up duration, BMI, hypertension, age > 75 years, transient ischemic attack or stroke, chronic obstructive pulmonary disease, heart failure, peripheral vascular disease. Antiarrhythmic medication is defined as class I antiarrhythmic medication, beta blockers, class III antiarrhythmic medication, calcium antagonists. Pulmonary vein isolation was performed maximal 365 days before or 14 days after the baseline visit. Abbreviations: AF = atrial fibrillation, BMI = body mass index. Abbreviations: AF = atrial fibrillation, BMI = body mass index, PVI = pulmonary vein isolation.
